# Ameliorative Effect of Thymoquinone and Thymoquinone Nanoparticles against Diazinon-Induced Hepatic Injury in Rats: A Possible Protection Mechanism

**DOI:** 10.3390/toxics11090783

**Published:** 2023-09-15

**Authors:** Walaa M. Nassar, Wafaa M. El-Kholy, Mamdouh R. El-Sawi, Nagi M. El-Shafai, Badriyah S. Alotaibi, Heba I. Ghamry, Mustafa Shukry

**Affiliations:** 1 Physiology Division, Zoology Department, Faculty of Science, Mansoura University, Mansoura 35516, Egypt; 2 Nanotechnology Center, Chemistry Department, Faculty of Science, Kafrelsheikh University, Kafrelsheikh 33516, Egypt; 3 Department of Pharmaceutical Sciences, College of Pharmacy, Princess Nourah bint Abdulrahman University, P.O. Box 84428, Riyadh 11671, Saudi Arabia; 4 Nutrition and Food Sciences, Department of Home Economics, Faculty of Home Economics, King Khalid University, P.O. Box 960, Abha 61421, Saudi Arabia; 5 Physiology Department, Faculty of Veterinary Medicine, Kafrelsheikh University, Kafrelsheikh 33516, Egypt

**Keywords:** thymoquinone, thymoquinone nanoparticles, diazinon, hepatic injury, oxidative stress, apoptosis, DNA damage

## Abstract

The health benefits of thymoquinone (TQ) have been a significant focus of numerous studies. However, more research is needed to ascertain whether its nano-form can effectively treat or prevent chronic diseases. In this study, we investigated how thymoquinone and its nanoparticles can mitigate liver damage induced by diazinon in male Wistar rats and explored the intracellular mechanisms involved. Forty-two Wistar male rats (*n* = 42) were randomly allotted into seven groups. Group 1 served as the control. Group 2 (vehicle) consisted of rats that received corn oil via a gastric tube daily. In Group 3 (TQ), rats were given a daily oral administration of TQ (40 mg/kg bw). Group 4 (thymoquinone nanoparticles, NTQ) included rats that received NTQ (0.5 mg/kg bw) orally for 21 days. Group 5 (DZN) involved rats that were administered diazinon (DZN, 15 mg/kg bw) orally. In Group 6 (TQ + DZN), rats first received TQ orally, followed by DZN. Group 7 (NTQ + DZN) consisted of rats receiving NTQ orally, then DZN. After 21 days of treatment, the rats were euthanized. After oral administration of DZN, liver enzymes were significantly elevated (*p* < 0.05). Additionally, there were noticeable increases in oxidative injury markers, such as nitric oxide, malondialdehyde, redox oxygen radicals, and overall increases in hydrogen peroxide and liver protein carbonyl concentrations. This was accompanied by the upregulation of apoptotic markers (Bax, caspase9, caspase 3, bax/Bcl2 ratio), inflammatory cytokines (TNF-α, IL-6), and DNA damage. There was also a noteworthy decrease (*p* < 0.05) in the activities of antioxidant enzymes and anti-apoptotic markers. However, the oral administration of thymoquinone or its nanoparticle form mitigated these diazinon complications; our histopathological findings corroborated our biochemical and molecular observations. In conclusion, the significant antioxidant properties of thymoquinone, or its nanoparticle form, in tandem with the downregulation of apoptotic markers and inflammatory cytokines, provided a protective effect against hepatic dysfunction caused by diazinon.

## 1. Introduction

The pervasive use of pesticides in agriculture significantly contributes to environmental pollution due to residual contamination. In particular, insecticides from the organophosphorus class are among the most frequently utilized [1]. Diazinon (DZN) is a highly popular organophosphate pesticide. Diazinon (DZN) is widely employed to eradicate pests such as cockroaches, silverfish, ants, and fleas. Its extensive use is not only limited to agricultural and horticultural settings but is also considered a critical component of pest management in crop production globally [2].

Diazinon functions as a potent inhibitor of acetylcholinesterase (AChE). When AChE is inhibited, acetylcholine accumulates in the synaptic cleft. After entering the body, diazinon can undergo oxidative degradation in the liver through the microsomal enzyme system. This process takes place in the presence of NADH and O_2_, resulting in the formation of diazoxon [3]. Diazoxon, an active oxygen metabolite of DZN, predictably induces a higher level of AChE inhibition than its parent molecule, as the latter does not directly inhibit AChE [4].

Furthermore, studies have shown that DZN can increase the generation of reactive oxygen species (ROS) and cause mitochondrial membrane damage. It also induces oxidative stress, oxidative modifications, lipid peroxidation, and DNA fragmentation in the genomic DNA of tissues or cells. These detrimental effects can lead to cardiotoxicity, neurotoxicity, genotoxicity, cytotoxicity, and apoptosis [5,6]. The pesticide DZN disrupts the body’s antioxidant defenses, leading to increased reactive oxygen species (ROS) generation [7].

Toxic material cadmium has been found to increase NO, MDA, H_2_O_2_, TNF-α, and IL-6 levels while decreasing SOD, CAT, GPx, and GST, leading to oxidative stress in common carp hearts [8]. Cui et al. [9] conducted a study on common carp kidneys exposed to cadmium and found apoptotic damage, specifically with Bax, caspase-9, and caspase-3. In another study, excess 4-tert-butylphenol induced apoptosis in the livers of common carp by increasing ROS, TNF-α, Bax, caspase-9, and caspase-3 levels [10]. In fish treated with tributyltin and chlorpyrifos, oxidative-stress-mediated apoptosis has been observed [11,12]. Furthermore, DZN poisoning can adversely impact the urinary, hematological, immunological, and pancreatic systems [13].

Antioxidants play crucial physiological roles in the body by suppressing oxidation. They help scavenge free radicals and convert them into less harmful byproducts. These antioxidants neutralize free radicals, mitigating their potential damage [14]. *Nigella sativa*, commonly known as black seed, is a herbaceous plant typically used as a natural food additive. This member of the Ranunculaceae family is native to the Middle East, central Europe, and western Asia. The seeds of this plant have been utilized for over two thousand years in traditional medicine, serving as both a preventative measure and a treatment method [15]. TQ, the primary active constituent extracted from the volatile oil of *Nigella sativa*, has been extensively studied. It has been documented to possess several pharmacological properties, including antioxidant activity and protective effects against hepatotoxins [16]. The pharmaceutical applications of TQ are extensive, encompassing the reduction of inflammation, protection against cancer, and neutralization of free radicals [17]. *Nigella sativa* seeds are utilized in treating a wide array of medical conditions. These include, but are not limited to, diarrhea, abdominal pain, dyslipidemia, asthma and coughs, headaches, dysentery, renal calculi, infections, obesity, back pain, hypertension, and various dermatological issues [18]. Nanotechnology holds immense global significance due to its myriad practical applications across diverse fields, including manufacturing, healthcare, biotechnology, agriculture, and various life sciences [19,20]. The physicochemical properties of NPs are unique and highly adaptable compared to those of bulk materials.

Due to cellular-level absorption and interactions, nanoparticles (NPs) pose an environmental hazard. This has created a divide among scientists as they weigh the potential drawbacks against the benefits of NPs [21]. The size, surface area, and concentration of generated NPs influence their bioactivity [22]. Nano-materials can readily traverse biological membranes, facilitating the targeted delivery of thymoquinone (TQ) to specific organs and tissues [23]. Therefore, nano-formulated thymoquinone (NTQ) will likely enhance absorption within the body [24]. In this study, we wanted to explore the intracellular mechanisms by which thymoquinone and its nanoparticle form protect the livers of male Wistar rats against diazinon toxicity. So, creating nano-sized delivery systems that can transport medications to their intended sites is one of the most exciting areas of study in the field of oral drug administration.

## 2. Materials and Methods

### 2.1. Chemicals and Preparation

Pesticide DZN was procured from the American Sigma Company, Ronkonkoma, NY, USA (code 454258-250MG), while TQ was obtained as a 99% pure analytical standard from Sigma-Aldrich, Schnelldorf, Germany. Pesticide DZN was dispersed in corn oil and orally administered to male albino rats at 15 mg/kg body weight [25]. TQ was dissolved in corn oil to prepare the required dose of 40 mg/kg body weight [26].

#### Fabrication of Thymoquinone Nanoparticles (NTQ)

A total of 2 g of thymoquinone was dissolved in 50 mL ethyl alcohol (95%), stirring at room temperature for 1 h. The suspension was ultrasonicated for 2 h; then, the drying process was conducted at 40 °C. The grinding method was performed for 5 h to form thymoquinone nanoparticles, evidenced by the various techniques.

**Figure 1:**
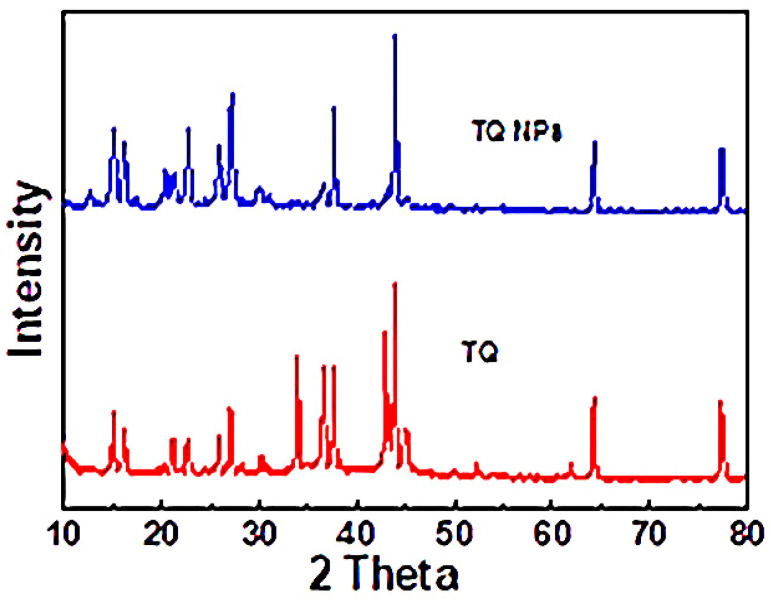
XRD patterns of the TQ nanoparticles.

Figure 1 illustrated the characteristic patterns of the synthesized TQ NPs, which were measured via the Shimadzu 6000, Kyoto, Japan. TQ appeared exhibited patterns at 12.4°, 14.95°, 16.1°, 21°, 22.8°, 25.8°, 26.9°, 30.4°, 33.9°, 36.65°, 37.66°, 43°, 43.8°, 45°, 50°, 52.2°, 62°, 64.3°, 77.4°, while the TQ NPs have characteristic XRD patterns at 12.4°, 15°, 16.1°, 20.4°, 21.2°, 26°, 27°, 30.1°, 36.5°, 37.8°, 43.8°, 44.9°, 64.3°, 77.4°. The results reveal that the patterns at 33.9°, 43°, 50°, and 52.2° disappeared while the patterns at 36.65 and 45 presented weak intensity; all of these were comparable to the patterns of NTQ. The results confirmed the formation of TQ NPs. From the Scherrer equation, the diameter of the TQ NPs was found to be 36 nm.

Zeta potential was measured using the zeta potential (Brookhaven) device at room temperature, and the experiment was performed in double-distilled water. The surface charges were studied using a diluted suspension of the tested NTQ, where the electrostatic stability of nanoparticles in double-distilled water were measured. The values ranged from ±30 to more than ±60 mV, and the value ±30 mV represented the dispersion stability of the NPs. When the voltage was more than ±60 mV, the stability was excellent [27]. NTQ had −38 nm in the aqueous solution, reflecting NTQ NPs’ stability. The powder of N-TQ was weighed and dissolved in corn oil to prepare a dose equal (to 40 mg/kg). The selected dose of N-TQ was according to the previous study [26].

### 2.2. Experimental Plan

The study involved 42 male white albino rats, each weighing on average between 90–100 g. These rats were procured from the Egyptian Institute for Vaccine and Serological Production in Helwan and housed in the Department of Physiology, Faculty of Veterinary’s animal facility at Kafrelsheikh University. The rats were kept in stainless steel cages with fresh wood chip bedding changed daily, under constant temperature and a 12 h light/dark cycle. Before the start of the experiment, all rats were acclimatized to their environment for two weeks. During this time, they had a regular diet and water ad libitum. After the acclimatization period, the animals were randomly divided into seven groups, each containing six rats. Control group: rats received oral saline. Corn oil group: for 21 days, rats were administered corn oil through a stomach tube daily. TQ-treated group: TQ (40 mg/kg bw) [26] was given orally to these rats once a day for 21 days. Nano-thymoquinone (N-TQ)-treated group: N-TQ (0.5 mg/kg bw) was given orally to rats once daily for 21 days. Pesticide-DZN-treated group: DZN (10 mg/kg) [28] was given orally to rats once daily for 21 days. TQ- and pesticide-DZN-treated group: rats were first orally administered TQ and DZN after one hour utilizing the same dose and regimen as employed in the third and fifth groups. N-TQ- and diazinon-treated group: rats were first orally administered N-TQ and DZN after one hour using the same dose and regimen used for the 4th and 5th groups. The experimental procedure was authorized by the Department of Zoology, Faculty of Sciences, Mansoura University, and was conducted in strict adherence to the guidelines from the National Research Council for the Care and Use of Laboratory Animals.

### 2.3. Sampling and Tissue Preparation

After the experiment, the rats were fasted overnight and then euthanized. Blood samples were collected, allowed to clot, and were then centrifuged at 4000 rpm for 15 min. The clear, non-hemolyzed serum was promptly isolated and stored in labeled Eppendorf tubes. These serum samples were frozen at −20 °C for future biochemical analyses. Liver samples were extracted from each rat, cleaned, weighed, and homogenized in a 10% weight/volume solution of distilled water. The supernatant from these samples was stored at −80 °C in labeled Eppendorf tubes until required for biochemical estimations. Additionally, liver tissue samples were preserved in a 10% neutral formalin solution for subsequent histopathological studies.

### 2.4. Biochemical Analysismin

The serum ALT and AST activities were measured using the NADH’s absorption coefficient at 340 nm and expressed as units per liter (U/L). One unit per liter was defined as the enzyme amount needed to oxidize one μmol/L of NADH per minute as a manufacturing guideline (Biodiagnostic Co., Giza, Egypt) [29]. ALP activity was determined using a kit from Biodiagnostic Co., Dokki, Giza, Egypt, following Belfield and Goldberg [30], in which p-nitrophenyl phosphate (pNPP) is a substrate for the alkaline phosphatase (ALP) enzyme, which, when dephosphorylated, turns yellow. The color intensity is measured at 405 nm. Lactate dehydrogenase (LDH) catalysis is the reduction of pyruvate by NADH; it was analyzed using kits provided by (Biodiagnostic Co., Giza, Egypt) [31]. Gamma-glutamyl transferase (GGT) activity was determined using kits provided by (Biodiagnostic Co., Giza, Egypt) following Szasz [32]. The method utilized a coupled-enzyme assay, where the gamma-glutamyl transferase (GGT) enzyme transferred the gamma-glutamyl group from the L-γ-Glutamyl-p-nitroanilide substrate. This resulted in the release of the chromogen p-nitroanilide, which was then measured at a wavelength of 418 nm. Total bilirubin level was determined calorimetrically using a kit (Diamond Diagnostics, Co., Cairo, Egypt), following Kaplan et al. [33]. Albumin was determined using a kit (Diamond Diagnostics Co., Cairo, Egypt), following Doumas et al. [34]. Using a colorimetric method, the MDA content of liver homogenate was measured with a kit from Biodiagnostic Co., Dokki, Giza, Egypt [35]. The process involves the colorimetric determination of a pink pigment product formed by reacting TBARS with thiobarbituric acid in an acidic medium at high temperatures. The resulting product is extracted with n-butanol, and its absorbance is measured at 532 nm—nitric oxide formation in liver homogenate by analyzing nitrite, a stable end product of NO oxidation. Liver nitrite concentration was determined using spectrophotometric analysis with the Griess reagent, which includes sulfanilamide, phosphoric acid, and N-1-naphthyl ethylenediamine dihydrochloride. This widely utilized method accurately measures nitric oxide formation in different biological samples, including liver homogenates, following Moshage et al. [36], using a kit from Biodiagnostic Co. (Dokki, Giza, Egypt).

H_2_O_2_ concentration was assayed following the manufacturing guide (Biodiagnostic Co., Dokki, Giza, Egypt). According to Pick [37], the tissue homogenate was prepared in normal saline and incubated with chilled methanol at 4 °C for 1 h. After centrifugation at 10,000× *g* for 30 min, the resulting supernatant was used to estimate the concentration of H_2_O_2_. For the assay, 100 μL of the supernatant was mixed with 100 μL of an assay solution containing phenol red, horse radish peroxidase, and potassium phosphate buffer in 0.9% NaCl. The reaction was initiated by adding 10 μL of 1.0 N NaOH, and the absorbance was measured at 600 nm. The amount of ROS in the liver was measured with an ELISA kit manufactured by AMSBIO, Co., Milton, UK, following Young et al. [38]. In brief, In the immunoassay technique, an anti-ROS antibody and a ROS-HRP conjugate were used. The buffer, sample, and conjugate were incubated together for 60 s in a pre-coated plate, followed by decanting, and washing of the wells. Next, a substrate was added to the wells, resulting in the formation of a blue-colored product through the reaction between the enzyme and substrate. To stop the reaction, a stop solution was used, which turned the solution yellow. The intensity of the resulting color was measured at 450 nm with a microplate reader (Anthos Labtec Instruments, Salzburg, Austria)., and it was found that the intensity was inversely proportional to the concentration of ROS. The concentration of ROS in each sample was determined by comparing the intensity of the resulting color to the standard’s concentration using a standard curve. A Quantitative Sandwich ELISA kit was used to determine the liver protein carbonyl (PC) concentration. The number of carbonyls in liver tissue was calculated by reacting 2,4-dinitrophenylhydrazine (DNPH) with protein carbonyls to form a Schiff base, which in turn, yields a hydrazone that can be spectrophotometrically examined using kits provided by (Cayman, Ann Arbor, MI, USA) [39].

Using kits supplied by Biodiagnostic Co. Dokki, Giza, Egypt, we determined the calorimetric activity of SOD, glutathione peroxidase (GPx), catalase (CAT), glutathione S-210 transferase (GST), and glutathione homogenate (GSH) in the liver. The method relies on the SOD enzyme’s ability to inhibit the phenazine-methosulphate (PMS)-mediated reaction of the nitro-blue tetrazolium dye, following Nishikimi et al. [40]. Glutathione peroxidase (GPx) activity was determined using the method developed by Paglia and Valentina [41]. In this method, the presence of glutathione reductase and NADPH leads to the immediate conversion of oxidized glutathione (GSSG) to its reduced form, accompanied by the oxidation of NADPH to NADP. The absorbance at 340 nm was measured to monitor the decrease.

Catalase (CAT): Following Aebi [42], the method uses catalase to decompose H_2_O_2_. After incubating the catalase-containing sample with a known concentration of H_2_O_2_ for one minute, the reaction is stopped with sodium azide. The remaining H_2_O_2_ is determined by the reaction between 4-aminophenazone (AAP) and 3,5-dichloro-2-hydroxybenzenesulfonic acid (DHBS), catalyzed by horseradish peroxidase (HRP). The resulting quinoneimine dye is measured at 510 nm. Glutathione S-transferase (GST): Following Habig et al. [43], the method depends on the catalytic reaction between CDNB (1-chloro-2 4-dinitrobenzene) and GSH (reduced glutathione) in the presence of GST (glutathione S-transferase). The absorbance at 340 nm was measured every 30 s for 3 min. Glutathione homogenate (GSH) in the liver was assessed following Beutler et al. [44], which involves the formation of a yellow compound through the reaction between DTNB (5,5′-dithiobis (2-nitrobenzoic acid)) and GSH. The intensity of the resulting chromogen was directly related to the concentration of GSH, and its absorbance can be measured at 405 nm, following the production manual. Liver homogenate total antioxidant capacity was determined using a colorimetric method (Biodiagnostic Co., Dokki, Giza, Egypt), following Koracevic et al. [39], in which the reaction between sample antioxidants and a provided amount of H_2_O_2_ was used to determine the total antioxidant capacity. The sample antioxidants neutralize some of the H_2_O_2_, and the remaining H_2_O_2_ is measured calorimetrically using an enzymatic reaction. The protein concentration was determined via the Bradford method [45].

### 2.5. Determination of Hepatic Bax, Bcl2, Caspase 3, and 9 Concentrations

Bax and Bcl-2 contents in the rat liver homogenate were determined quantitatively using Rat Bax and Bcl-2 ELISA kits (EIAab, Science Co., Ltd., Wuhan, China) and (USCN Life Science Inc., Wuhan, China), respectively. In brief, the assay involved dispensing 50 μL of samples into 96-well plates coated with rat, Bax, or Bcl-2 antibodies. After incubation at room temperature and washing, 100 μL of Streptavidin-HRP was added and incubated for 30 min at 37 °C. A colorimetric assay was performed using 3,3,5,5-tetramethylbenzidine (TMB) as the chromogen (100 μL/well). The reaction was stopped after 10 min by adding the stop solution (100 μL/well), and the resulting absorbance at 450 nm was measured.

Caspase-9 and caspase-3 concentrations in liver homogenate were determined quantitatively using Rat caspase-3 and caspase-9 ELISA kits supplied by My BioSource (San Diego, CA, USA) and CUSABIO (Baltimore, MD, USA), respectively. To assess Caspase-3 activity, 100 μL of cell lysates were added to each well and incubated for 2 h at 37 °C. After incubation, the wells were washed with 200 μL of washing buffer 1×. Then, 100 μL of biotinylated caspase-3 rabbit antibody was added and incubated for 1 h at 37 °C. Following this, 100 μL of HRP-conjugated second antibody was added and incubated for 30 min at 37 °C. Next, 100 μL of TMB substrate was added and incubated for 10 min at 37 °C. Finally, 100 μL of stop solution was added, and the absorbance at 450 nm was measured. For Caspase-9 activity, the liver extracts were suspended in a lysis buffer and centrifuged at 20,000× *g* for 20 min. The remaining pellet was then suspended in a caspase buffer. Caspase-9 specific substrate was added, and the samples were incubated for 4 h at 37 °C. Finally, the absorbance measured at 405 nm was measured.

### 2.6. Determination of Hepatic Inflammatory Cytokines

We used rat ELISA kits from Boster Biological Technology, specifically the Picokine™ kits, USA, to quantitatively assess TNF-α in the liver homogenate. For IL-6, we used kits from the Norcross, GA, USA producer. All measurements were taken following the guidelines provided by the producer.

### 2.7. Determination of DNA Damage in the Liver via Comet Assay

The comet analysis was performed following Singh et al. [46]. As soon as the animal was euthanized, a small piece of liver was removed and diced finely before adding 1 mL of cold PBS with 20 mM EDTA/10% DMSO to make a cellular suspension. A total of 10 microliters of suspension were encased in 75 microliters of 1% low-melting-point agarose and then dispersed on a slide coated with 1% normal agarose. After allowing the agarose to set for 5–10 min at 4 °C, the slides were submerged in lysis solution (2.5 M NaCl, 100 mM EDTA, ten mM Trizma base, NaOH to pH 10.0) with newly added 1% Triton X-100 and 10% DMSO overnight at 4 °C.

Next, the buffer reservoirs were filled with freshly prepared pH > 13 electrophoresis buffer containing 300 mM NaOH and one mM EDTA for 20 min, and the slides were placed side by side on the horizontal gel box for 30 min of electrophoresis at 24 V at room temperature. Before staining with 80 μL of ethidium bromide (20 μg/mL), the slides were washed three times for five minutes in a neutralization buffer containing 0.4 M Tris-HCl, pH 7.5. DNA damage can be seen under a fluorescence microscope when ethidium bromide-stained DNA is examined with a 40× lens.

### 2.8. Histopathological Studies

Sections of the liver were cut at 5–7 μm in thickness using a microtome and stained with eosin and hematoxylin after being accurately fixed in a 10% neutral formalin solution, dehydrated in an escalating series of ethanol, cleaned in xylene, and embedded in paraffin wax. Histopathological alterations were identified by visually inspecting the stained sections under a light microscope [47].

### 2.9. Statistical Analysis

One-way analysis of variance (ANOVA) and the Newman-Keuls multiple comparison tests were used to analyze the data using GraphPad Prism 8.0 software. Statistical significance was assumed for *p* values less than *p* < 0.05.

## 3. Results

### 3.1. Liver Function Parameters

Our results reveal that serum levels of ALT, AST, GGT, ALP, and LDH were significantly increased (*p* < 0.05) in the DZN-treated rats compared to the other treated groups. In addition, the TQ and N-TQ groups co-treated with DZN showed decreased ALT, AST, GGT, ALP, and LDH levels. However, the serum total bilirubin was also significantly increased in the DZN-treated rats compared to the other treated groups; co-treatment with TQ and N-TQ significantly decreased total bilirubin. However, the serum total protein and albumin were reduced considerably in the DZN-treated rats compared to the other groups, while TQ and N-TQ rats co-treated with DZN showed significantly increased total protein and albumin compared to DZN-treated groups. The effect was more pronounced in the N-TQ group compared to the TQ-treated group, as shown in Table 1.

### 3.2. The Markers of Oxidants/Antioxidants and Cytokines

**Figure 2:**
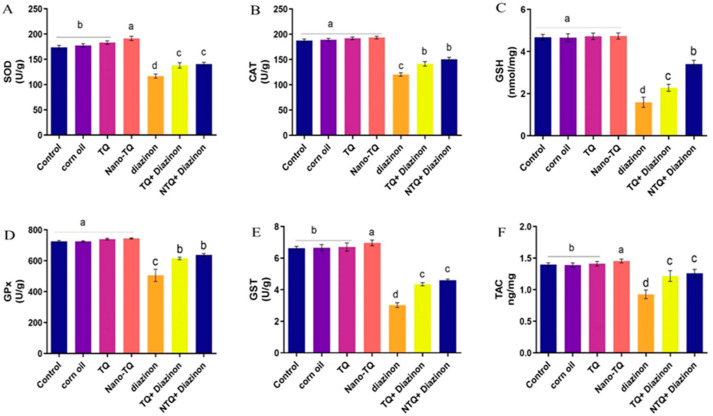
The impact of TQ and NTQ antioxidant status on hepatic tissue contents of DZN-treated rats. (**A**) SOD, (**B**) CAT, (**C**) GSH, (**D**) GPx, (**E**) GST, and (**F**) TAC. The data are presented as mean ± SEM. ^a–d^ Different superscript letters indicate a statistically significant difference between means at *p* < 0.05.

As shown in Figure 2, the SOD, CAT, GSH, GPx, GST, and TAC levels were notably reduced in the DZN-treated groups compared to other treated ones. In addition, the TQ and N-TQ groups co-treated with DZN exhibited a notably improved level compared to the DZN-treated rats. The improvement was more apparent in the NTQ-treated DZN group than in the TQ-treated DZN-treated group.

**Figure 3:**
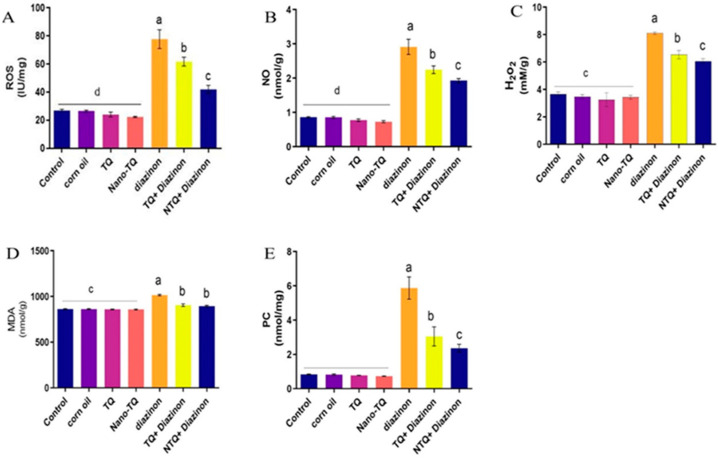
Effects of TQ and N-TQ on the oxidant status in hepatic tissue contents of DZN-treated rats. (**A**) ROS International unit/mg (IU/mg), (**B**) NO (nmol/g), (**C**) H_2_O_2_ (mM/g), (**D**) MDA (nmol/g), (**E**) PC (nmol/mg). Data are shown as mean ± SEM. ^a–d^ means carrying different superscript letters significantly differ at *p* < 0.05.

The results reveal that the concentration of ROS, NO, H_2_O_2_, MDA, and PC in the DZN group was much greater than in the control groups. The TQ and NTQ groups co-treated with DZN demonstrated a substantial decrease in their quantities compared to the DZN-treated rats. The improvement was more apparent in the NTQ group treated with DZN than in the TQ group treated with DZN (Figure 3).

**Figure 4:**
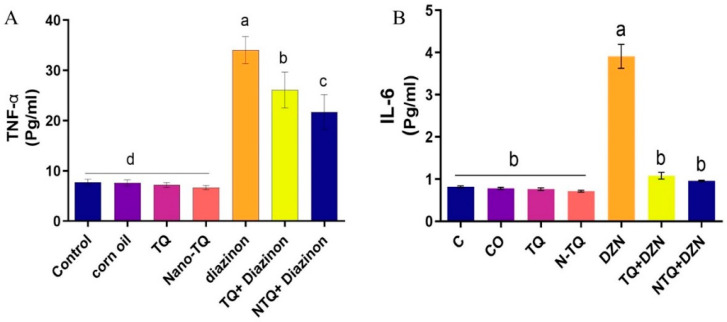
Impact of TQ and N-TQ on cytokines (**A**) TNF-α and (**B**) IL-6 status in hepatic tissue contents of DZN-treated rats. The data are presented as Mean ± SEM. ^a–d^ means with distinct superscript letters differ statistically substantially at the *p* < 0.05.

According to the findings, the concentration of TNF-α and IL-6 in the DZN-treated rats was notably higher than in the other groups. The TQ and N-TQ groups co-treated with DZN exhibited a marked reduction in concentration in the DZN-treated group. The improvement was more apparent in N-TQ group treated with DZN than in the TQ group treated with DZN, as seen in Figure 4.

### 3.3. Apoptotic Markers

**Figure 5:**
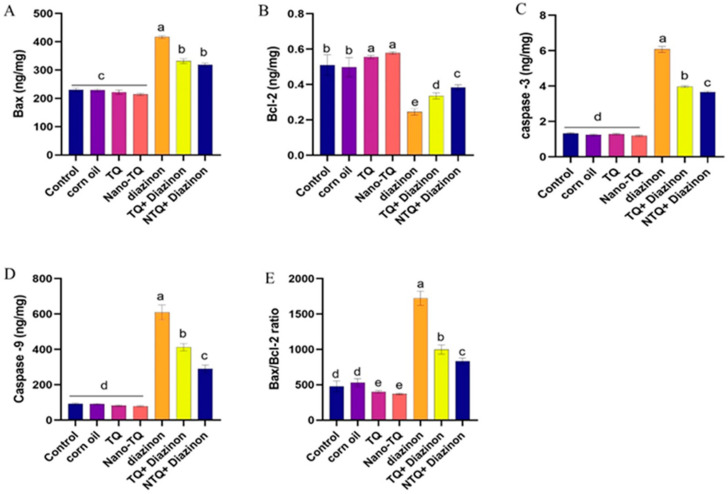
Impact of TQ and NTQ on apoptosis marker status in hepatic tissue contents of DZN-treated rats. (**A**) Bax, (**B**) Bcl2, (**C**) Caspase-3, (**D**) Caspase-9, (**E**) Bax /Bcl2 ratio. The data are presented as mean SEM. ^a–e^ means with distinct superscript letters differ statistically substantially at the *p* < 0.05.

The results revealed that the DZN-treated group had considerably higher levels of Bax, caspase-3, caspase-9, and the Bax/Bcl-2 ratio than the other groups. The TQ and N-TQ groups co-treated with DZN demonstrated a meaningful decrease in their quantities compared to the DZN-treated group. The improvement was more apparent in the N-TQ group treated with DZN than in the TQ group treated with DZN. While the Bcl-2 concentration considerably declined in the DZN-treated rats compared to the other treated groups, its level was significantly improved in both The TQ and N-TQ groups co-treated with DZN. The improvement was more apparent in N-TQ group treated with DZN than in the TQ group treated with DZN treated, as shown in Figure 5.

### 3.4. DNA Damage

**Figure 6:**
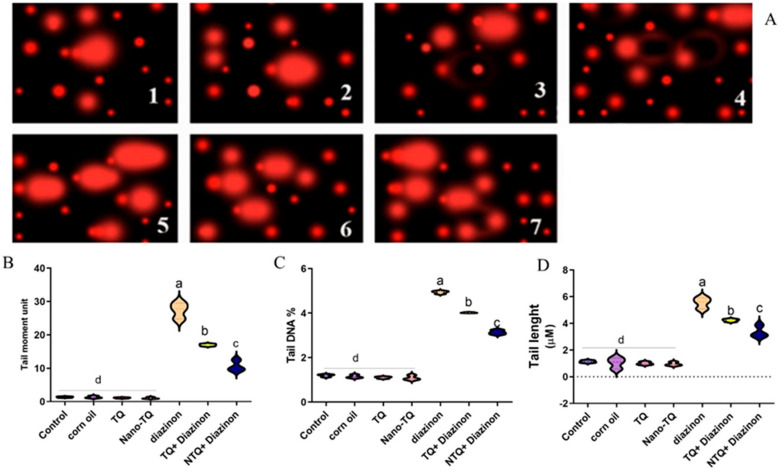
(**A**) Fluorescent photomicrograph (40×) of the influence of TQ and NTQ on DNA damage percentage as measured via comet test in rat liver tissues treated with DZN. (**B**) Tail moment unit. (**C**) Tail DNA%. (**D**) Tail length. The data are presented as mean SEM. ^a–d^ means with distinct superscripts are statistically different at *p* < 0.05.

The results show that the DZN group had considerably higher levels of tail DNA, tail length, and tail moment than the control group and the other treatment groups. The TQ and NTQ groups co-treated with DZN demonstrated a meaningful decrease in these levels compared to the DZN-treated rats. The improvement was more apparent in N-TQ group treated with DZN than in the TQ group treated with DZN; the enhancement was more evident in the NTQ group than in the TQ-treated rats, as shown in Figure 6.

### 3.5. Histological Analysis

**Figure 7:**
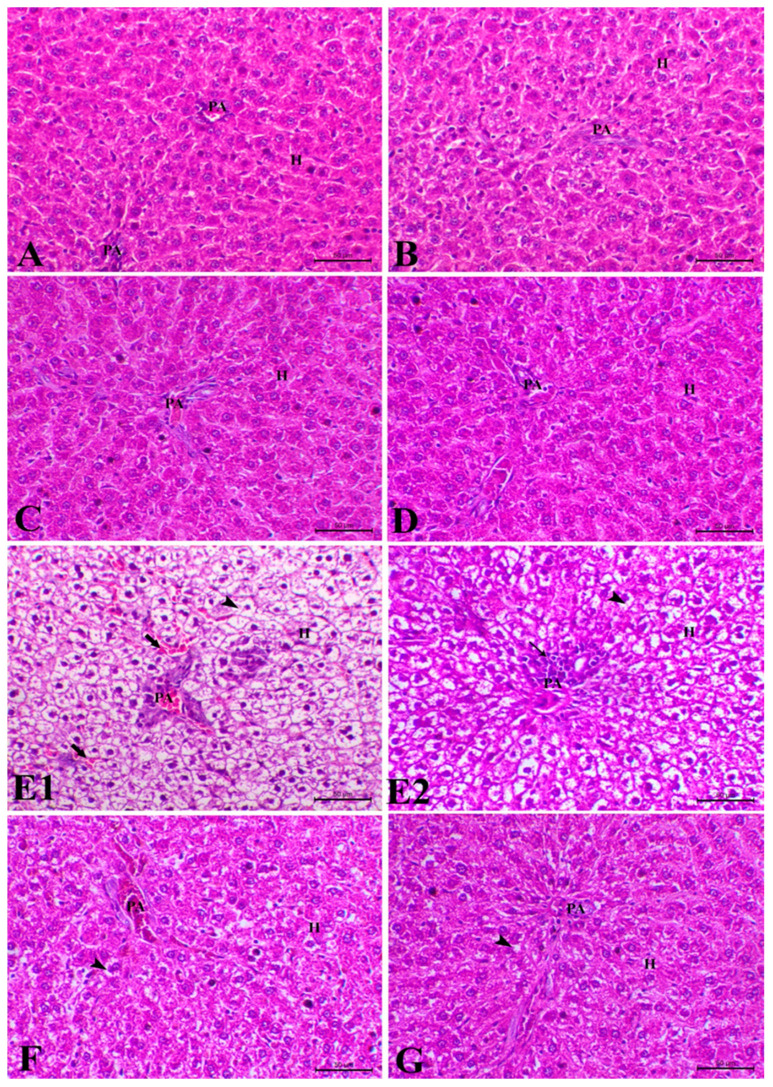
Photomicrograph of hepatic tissue sections stained with H&E. (**A**) The liver of control rats displayed a typical appearance of the central vein with normal epithelial cells, hepatocytes (H) around the portal area (PA), blood sinusoids, and Kupfer cells. (**B**) The liver of sham rats showed a normal appearance of the central vein with normal epithelial cells, hepatocytes (H) around the portal area (PA), blood sinusoids, and Kupfer cells. (**C**) Liver of normal animals treated with TQ showed normal appearance of the central vein with normal epithelial cells, hepatocytes (H) around the portal area (PA), blood sinusoids, and Kupfer cells. (**D**) Liver of normal animals treated with NTQ showed typical appearance of the central vein with normal epithelial cells, hepatocytes (H) around the portal area (PA), blood sinusoids, and Kupffer cells. (**E1**,**E2**) Liver of positive animal treated with diazinon showed severe periportal hepatic vacuolation, hepatocyte around the portal area (PA) (arrowheads), nuclear pyknosis, congested central vein, hemorrhage in the partial area and blood sinusoid, dilatation and congestion of blood sinusoids (thick arrows) along with mono nuclear cell infiltration (thin arrow). (**F**) Liver of diseased animals treated with TQ showed a decrease in the periportal vacuolation around the portal area (PA) (arrowhead), normal portal area, and blood sinusoid. (**G**) The liver of diseased animals treated with NTQ showed an apparent decrease in the periportal vacuolation around the portal area (PA) (arrowhead), normal portal area, and blood sinusoid. H&E, ×200, bar = 50 µm.

The livers of the control, corn oil, TQ, and N-TQ groups showed the usual central vein and the epithelial cells, normal hepatocytes, blood sinusoids, and Kupffer cells. The DZN-treated group showed severe periportal hepatic vacuolation nuclear pyknosis, congested central vein, bleeding in the partial area and blood sinusoid, dilatation, and congestion of blood sinusoids and cell infiltration. In addition, the TQ-treated DZN rats and the N-TQ-treated DZN rats showed decreased periportal vacuolation with normal portal area and blood sinusoid. Still, the improvement was evident in the N-TQ-treated DZN rats, as shown in Figure 7.

## 4. Discussion

The widespread use of pesticides in agriculture has led to growing environmental concerns due to the resulting contamination. One common type of pesticide used for pest control is organophosphorus insecticides [48]. Long-term exposure to organophosphorus can occur through agricultural activities, household use, and the consumption of contaminated food and water. Traces of organophosphorus have been detected in various food items, including cereals, fruits, and vegetables, as well as in water and soil [49]. One of the most extensively utilized organophosphorus chemicals in agriculture is the pesticide, DZN [50]. Various tissues, particularly the kidney and liver, metabolize pesticide DZN and facilitate its rapid elimination through processes of oxidation and hydrolysis [51]. Furthermore, it inhibits the enzyme acetylcholinesterase (AChE), which plays a crucial role in maintaining the health of nervous system [52]. Moreover, research indicates that pesticide DZN can elevate the production of reactive oxygen species (ROS), cause mitochondrial membrane damage, induce oxidative stress, oxidative modifications, lipid peroxidation, and DNA fragmentation in the genomic DNA content of tissues and cells. These effects can lead to cardiotoxicity, neurotoxicity, genotoxicity, cytotoxicity, and apoptosis [53].

TQ, the primary phytochemical constituent of *Nigella sativa*, boasts numerous health benefits and is utilized in various natural medicinal applications. Its effects include antioxidant, anti-inflammatory, immune-stimulating, antibacterial, hypoglycemic, anti-arthritic, and hepatoprotective properties [54]. TQ’s potent antioxidant and anti-inflammatory properties contribute significantly to the reduction of hepatotoxicity [55].

Serum levels of ALT, AST, GGT, ALP, LDH, and total bilirubin (TB) were all found to be significantly higher in rats treated with DZN for 21 days; our finding was in line with Abdel-Daim et al. [56]; they found that DZN-induced oxidative stress may be responsible for the elevation of liver enzymes (ALT, AST, GGT, Alp). This is because the DZN damaged hepatocytes, which leads to the leakage of these enzymes into the systemic circulation [57]. Thus, DZN-induced enzyme leakage from the mitochondrial membrane may account for the elevated level of blood LDH. In addition, the toxic nephrosis impact of DZN can cause significant protein loss in the urine, leading to hypoalbuminemia, which was supported by Karimani et al. [58]. Serum levels of hepatic enzymes such as ALT, AST, ALP, GGT, and TB dropped while levels of TP and albumin rose to almost control levels after being treated with TQ and NTQ. These findings corroborate those of Al-Malki and Sayed [59], who reported TQ’s hepatoprotective effect. It was hypothesized that TQ prevented intracellular antioxidant depletion and hepatotoxicity caused by blocking the covalent attachment of free radical products to intracellular macromolecules such as lipids, proteins, and DNA [60]. The higher cellular permeability of NTQ, when it interacts with the lipid membrane of a hepatocyte, may explain the pronounced improvement effect seen in the NTQ-treated group, of alleviating the harmful impact of the DZN-induced hepatic injury [61]. ROS are detrimental because of a mismatch between their production and destruction, which disrupts normal cellular physiology and causes a variety of disease states [62]. DZN has a strong binding affinity for phospholipid bilayers found in all biological membranes. In the liver of rats, it disrupts the transit of mitochondrial membranes [63]. The present study showed that MDA, ROS, NO, H_2_O_2_, and protein carbonyl (PC) increased significantly after oral administration of DZN, accompanied by significant decreases in SOD, CAT, GST, GPx, and TAC levels; these findings were in harmony with those of Ajibade et al. [64], who reported elevations in renal tissue injury markers that are associated with oxidative stress and increased ROS production as a result of DZN toxicity [65]. Free radical generation by DZN in rat liver tissue was shown to enhance MDA levels, as reported by SH AQ [66]. Organophosphates were demonstrated to increase NO production by increasing NO synthase activity [67]. Protein carbonyl (PC) is the most widely used marker of protein oxidation and the most universal signal for oxidative stress [68].

DZN inhibits SOD activity, which increases ROS accumulation and tissue damage [69]. Conversely, oxidative stress markers were significantly reduced after oral administration of TQ and NTQ. Previous investigations also yielded comparable results, as demonstrated by El-Magd et al. [70]. TQ’s antioxidant capabilities come from its ability to scavenge ROS and block the production of 5-hydroxyeicosatetraenoic acid and 5-lipoxygenase, two compounds that may have a role in illness [71].

Our data revealed that TQ upregulates SOD, GPX, CAT, and GST expression in rat livers, which may be responsible for increased antioxidant levels and which may activate intracellular antioxidants, as reported by [61,72]. This suggests that TQ’s direct antioxidant activity and activation of endogenous antioxidants may contribute to its ability to decrease organophosphate toxicity [73]. In addition, TQ neutralizes the free radicals produced by the superoxide, hydroxyl [74]. Previous research has shown that TQ can prevent NO generation by blocking the synthesis of iNOS proteins and the expression of iNOS genes in rats [75]. TQ might neutralize free radicals and protect the antioxidant enzymes CAT, GPx, and GST from deactivation [76].

The present investigation demonstrated that Bax, Caspase-3, Caspase-9, and the Bax/Bcl-2 ratio were significantly increased after oral administration of DZN, while Bcl-2 decreased considerably. The findings are consistent with these results [77]. DZN was found to increase the amount of apoptotic cells in hepatic tissues and produce toxic damage to hepatocytes [78]. DZN causes mitochondrial damage by increasing membrane permeability and generating ROS [79]. Mitochondrial apoptosis is strongly triggered by exposure to different cytotoxic and stress-related stimuli, including ROS. Caspase-9 and its effectors, Caspases 3 and 7, are activated when mitochondrial injury releases cytochrome c, leading to DNA breakage and cells dying [80].

The pro-apoptotic protein, Bax, is countered by the anti-apoptotic protein, Bcl-2, which prevents mitochondrial membrane permeability [81]. Caspase-3, Caspase-9, Bax, and the Bax/Bcl-2 ratio inhibited, while TQ and NTQ boosted Bcl-2 in the current study. These results are consistent with those of Hamad and Kadhem [82]. This might be because TQ suppresses organophosphates and tumor necrosis factor (TNF-α), consequently inhibiting apoptosis.

Our data showed that TQ treatment decreased the Bax/Bcl-2 ratio in the liver, both in terms of protein and mRNA. Caspase-3, -8, and -9 activation were also reported to be reduced by TQ [83]. Caspase-3 expression, an indicator of apoptosis, was also shown to be suppressed by TQ, as was previously reported that caspase-3 expression was also shown to be stifled by TQ [84]. In addition, the molecular analysis indicated that TQ inhibits apoptosis by showing a significant drop in Caspase-3 and Bax gene mRNA expression by inhibiting the release of cytochrome c, a cornerstone of the intrinsic apoptotic cascade [78].

In line with Birdane et al. [64], our study revealed that TNF-α and IL-6 were significantly elevated in DZN-treated rats; this elevation may be attributable to the positive correlation between oxidative stress and TNF-α expression [85]. In addition, DZN intoxication may promote TNF-α expression directly or indirectly through oxidative stress. TNF-α can directly harm hepatic cells. The inflammatory response was triggered by the oxidative stress created by the DZN application [86], or because DZN induced a direct upregulation of pro-inflammatory cytokine mRNA expression [87]. In contrast, the present investigation demonstrated that TQ and NTQ significantly reduced TNF-α and IL-6 as inflammatory markers when given orally to male Wistar albino rats. The findings are in line with those of El-Magd et al. [70].

The observed trend explains TQ’s potential to curb IL-6 by blocking ROS production [88]. In addition, TQ contains anti-inflammatory properties [59]. TQ reduced the production of several inflammatory mediators in the body, such as interleukins and TNF-α [73]. These findings highlight that TQ can treat and prevent inflammation by suppressing inflammatory cytokines. The present study demonstrated that oral administration of DZN caused dramatic DNA damage in adult Wistar rats. These findings are supported by Pehlivan and Durdaği [89]. Organophosphorus compounds with alkylating characteristics may be responsible for this deterioration. DNA damage likely involves the direct or indirect alkylation of bases, either by a base-modifying enzyme or a protein [89]. DNA oxidative damage was also observed, with DZN increasing the frequency of basic sites in DNA [90]. It is also possible that DZN increases the production of reactive free radicals, which can exacerbate DNA damage [91]. The mechanisms behind this damage and the resulting cellular effects: DZN has been shown to damage DNA and cause cellular dysfunction by forming DNA adducts and lesions, single-strand and double-strand DNA breaks, and DNA and protein inter- and intra-cross-links [92], In addition, DZN exposure increases the occurrence of micronuclei (genetic damage) due to strand breaks in DNA and chromosome aberrations caused by oxidative stress [93]. However, TQ and NTQ treatment significantly protected against DNA damage compared to the DZN-treated group. TQ’s ability to scavenge ROS and restore the rat antioxidant system may be responsible for the enhancement [94].

Furthermore, Gore et al. [95] reported that TQ’s antioxidant action and upregulation of the Nrf2 pathway provide organ protection. It was also reported that TQ increased the expression of genes that help detect and repair damaged and fragmented DNA. This impact may give more defense against DNA damage, leading to higher rates of cellular survival [96]. When examining the histopathological alterations brought on by DZN administration, the results showed nuclear pyknosis, congestion of the central vein, partial area and blood sinusoid hemorrhage, dilatation and congestion of blood sinusoids, mononuclear cell infiltration, and activated Kupffer cells. In line with these findings, Mahmoud [1] reported that the ROS production generated by DZN is responsible for these effects [13]. DZN dramatically increased MDA and PC, correlated with its cytotoxic effects on hepatic cells [68]. Fat alteration and hepatocyte necrosis can occur due to DZN’s ability to inhibit protein synthesis and hydrolysis [97]. TQ and NTQ, when taken orally, however, noticeably enhanced liver function; these findings are consistent with El-Magd et al. [70], TQ’s direct antioxidant impact and indirect ROS-scavenging activity contribute to its ability to lower oxidative stress [98]. To target drugs efficiently, they must be transported to the desired location without compromising their functions and structure [99]. In recent years, nanoparticles have become increasingly important in the development of drug and gene delivery systems. Specifically, mesoporous silica nanoparticles (MSNs) have shown advantages over other nanocarriers in delivering cancer treatment drugs. These nanoparticles have the ability to load a large number of drugs and can be modified to enhance their therapeutic effects. Additionally, MSNs can overcome multidrug resistance in cancer cells and have been used for gene delivery as well. Multifunctional systems based on MSNs are also being explored for controlled drug and gene delivery [100]. Using MSNs to deliver telmisartan enhances its dissolution rate and bioavailability compared to the pure drug powder. Entrapment of telmisartan in the pores of MSNs improves its oral absorption by enhancing permeability and reducing drug efflux [101]. The use of nanomaterials, particularly selenium nanoparticles, offers a solution for tackling antimicrobial resistance. These nanoparticles demonstrate antimicrobial efficacy against viruses, bacteria, and chronic diseases. One notable advantage is their straightforward and cost-effective synthesis, coupled with their minimal harm to eukaryotic cells [102]. In addition, nano-materials can easily cross biological membranes and deliver TQ to specific locations in the body in a sustained fashion [103]. So, N-TQ gives us the best results.

## 5. Conclusions

The present study has proven that TQ and its nanoparticle form, NTQ particles, have a hepatoprotective effect against DZN-induced hepatic injury. The mechanisms underlying this effect may involve counteracting DZN-induced oxidative injury markers, such as the significant increase in NO, ROS, H_2_O_2_, MDA, and protein carbonyl, as well as an increase in apoptotic markers. Thymoquinone and its nanoparticle form were found to mitigate the complications caused by diazinon through their powerful antioxidant, anti-inflammatory, and anti-apoptotic effects, in addition to reducing DNA damage. These findings suggest that NTQ particles can easily cross biological membranes and sustainably deliver thymoquinone to specific locations in the body. Overall, the study highlights the hepatoprotective potential of thymoquinone and its nanoparticle form, with NTQ particles demonstrating the best results.

## Figures and Tables

**Table 1:** Protective impacts of TQ and NTQ on diazinon-induced liver dysfunction in rats.

Group	Control	Corn Oil	TQ	NTQ	DZN	TQ + DZN	NTQ + DZN
AST (U/L)	41.50 ± 1.94 ^c^	40.75 ± 2.72 ^c^	39.50 ± 4.13 ^c^	34.73 ± 1.9 ^d^	143.00 ± 7.15 ^a^	60.75 ± 4.37 ^ab^	57.48 ± 4.44 ^b^
GGT (U/L)	15.55 ± 0.70 ^c^	15.50 ± 0.66 ^c^	13.63 ± 1.12 ^c^	13.38 ± 1.0 ^c^	26.63 ± 2.1 ^a^	18.78 ± 0.56 ^b^	17.18 ± 0.33 ^b^
ALT (U/L)	50.75 ± 3.79 ^c^	51.05 ± 4.11 ^c^	50.38 ± 4.04 ^c^	48.53 ± 3.9 ^c^	74.75 ± 2.3 ^a^	60.75 ± 2.39 ^b^	55.00 ± 3.94 ^b^
ALP (U/L)	268.80 ± 4.09 ^c^	269.00 ± 5.6 ^c^	266.50 ± 5.6 ^c^	257.00 ± 2.2 ^d^	485.30 ± 2.7 ^a^	438.30 ± 14.1 ^ab^	387.30 ± 5.3 ^ab^
LDH (U/L)	230.8 ± 1.65 ^c^	229.3 ± 1.65 ^c^	228.3 ± 1.25 ^c^	225.6 ± 1.7 ^c^	519.5 ± 4.8 ^a^	434.5 ± 11.53 ^ab^	410.8 ± 15.90 ^ab^
Total protein (mg/dL)	6.80 ± 0.20 ^a^	6.71 ± 0.20 ^a^	6.71 ± 0.29 ^a^	6.90 ± 0.16 ^a^	4.27 ± 0.07 ^d^	5.51 ± 0.14 ^c^	6.10 ± 0.38 ^b^
Albumin (mg/dL)	3.64 ± 0.158 ^a^	3.49 ± 0.070 ^a^	3.46 ± 0.160 ^a^	3.68 ± 0.22 ^a^	2.51 ± 0.17 ^c^	3.35 ± 0.176 ^b^	3.53 ± 0.175 ^b^
Total bilirubin (mg/dL)	0.59 ± 0.02 ^c^	0.55 ± 0.03 ^c^	0.52 ± 0.02 ^c^	0.42 ± 0.05 ^c^	1.55 ± 0.03 ^a^	1.06 ± 0.10 ^ab^	1.05 ± 0.10 ^ab^

The data are displayed as mean ± SEM, where ^a–d^ denotes statistically significant differences between means in the same row at the *p* < 0.05.

## Data Availability

Upon request.
